# Modern molecular approaches for analyzing microbial diversity from mushroom compost ecosystem

**DOI:** 10.1007/s13205-015-0289-2

**Published:** 2015-03-18

**Authors:** Pavan Kumar Agrawal, Shruti Agrawal, Rahul Shrivastava

**Affiliations:** 1 Department of Biotechnology, G. B. Pant Engineering College, Ghurdauri, Pauri, Uttarakhand India; 2 Department of Microbiology, Sai Institute of Paramedical and Allied Sciences, Dehradun, Uttarakhand India; 3 Department of Biotechnology and Bioinformatics, Jaypee University of Information Technology, Waknaghat, Solan, Himachal Pradesh 173234 India

**Keywords:** Microbial diversity, Cultivation dependent approach, Non-cultivable approach, Sequencing

## Abstract

Biosphere is a store house of various microorganisms that may be employed to isolate and exploit microbes for environmental, pharmaceutical, agricultural and industrial applications. There is restricted data regarding the structure and dynamics of microbial communities in several ecosystems because only a little fraction of microbial diversity is accessible by culture methods. Owing to limitations of traditional enrichment methods and pure culture techniques, microbiological studies have offered a narrow portal for investigating microbial flora. The bacterial community represented by the morphological and nutritional criteria failed to provide a natural taxonomic order according to the evolutionary relationship. Genetic diversity among the isolates recovered from mushroom compost has not been widely studied. To understand genetic diversity and community composition of the mushroom compost microflora, different approaches are now followed by taxonomists, to characterize and identify isolates up to species level. Molecular microbial ecology is an emerging discipline of biology under molecular approach which can provide complex community profiles along with useful phylogenetic information. The genomic era has resulted in the development of new molecular tools and techniques for study of culturable microbial diversity including the DNA base ratio (mole% G + C), DNA–DNA hybridization, DNA microarray and reverse sample genome probing. In addition, non-culturable diversity of mushroom compost ecosystem can be characterized by employing various molecular tools which would be discussed in the present review.

## Introduction

Mushroom compost is an interesting example of ecosystem with complex spectrum of microbial diversity. It is a rich reservoir of microbial types, comprising mesophilic and thermophilic bacteria, fungi and actinomycetes (Rawat and Johri 2014). The mesophilic microflora forms the pioneer community, while thermophiles represent the climax community. Microbial biodiversity of compost is important because it takes part in breakdown of organic material. The fast-growing *Pseudomonads* and *Arthrobacter* constitute the pioneer flora (Hayes et al. 1969; Stanek 1972) that rapidly degrades high concentration of organic matter, while *Bacilli* have been reported to be the dominant bacteria of not only mushroom compost (Libmond et al. 1995; Agrawal et al. 2011) but also of other compost ecosystems (Strom 1985).

Microbial community succession during composting is a classical example of how the growth and activity of one group of organism creates the condition necessary for the growth of others (Agrawal 2014). Several generations of microorganisms succeed each other during composting, wherein each crop of microbial form utilizes the available material in the substrate as also the cellular component of its predecessors for growth, spread and sustenance. The microbial abundance, composition and activity, changes substantially during composting; and compost maturity could be correlated with high microbial diversity and low activity (Ryokeboer et al. 2003). The study of community structure and diversity has been instrumental in manipulating the compost environment to quicken the composting process and to improve compost quality (Peters et al. 2000).

Approaches to characterize and classify microbial communities by cultivation methods have switched to the molecular and genetic level. Cultivation-based techniques have allowed merely a glimpse of microbial diversity as only an estimated 1 % of the naturally occurring bacteria has been isolated and characterized so far (Muyzer 1999). Chandna et al. (2013) reported *Kocuria*, *Microbacterium*, *Acidovorax* and *Comamonas* from agricultural byproducts compost using culture-based approaches. However, to understand better the nature of bacterial communities associated with compost, culture-independent molecular approaches based on sequencing of 16S rRNA genes were used to describe the complete bacterial community composition; new genera *Kocuria*, *Microbacterium*, *Acidovorax* and *Comamonas* have been identified from the compost which can be used as compost inoculants for accelerating the composting process.

Modern molecular approaches have revealed an extraordinary diversity of microorganisms, most of which are yet uncharacterized because of non-culturable nature of microorganisms. This poses a great challenge to microbial ecologists. How could one compare the microbial diversity of different environments when vast majority of microbial taxa is usually unknown? Bohannan and Hughes (2003) have reported three statistical approaches to analyze microbial diversity such as parametric estimation, non-parametric estimation and community phylogenetics which are proving to be promising tools to meet this challenge. Parametric and non-parametric estimation approaches are used to compare operational taxonomic unit (OUT) richness among environments, while phylogenetic approach compares evolutionary diversity of organisms among environments. Microbial biodiversity describes complexity and variability among microorganisms at different levels of biological organization. It includes genes, species, ecosystems, evolutionary and functional processes that link them (www.for.gov.bc.ca/pab.publctns/glossary/b.htm). Microbial diversity constitutes an extraordinary reservoir of life in the biosphere that has only just begun to be explored and understood (Jain et al. 2005). Huston (1994) had reported that highest diversity occurred in communities where many different species were present (richness) in relatively equal abundance (evenness). Microorganisms represent a rich repertoire of molecular and chemical diversity in nature as they comprise the most diverse form of life. Torsvik et al. (2002) have reported that more than 99 % bacteria from environmental samples remain ‘unculturable’ in the laboratory. Many of these unculturable bacteria represent new phylotypes, families and divisions in domains bacteria and archaea. ‘Unculturable’ bacterial diversity presents a vast gene pool for biotechnological exploitation and poses a major challenge to understand their phylogenetic relationship and ecological significance. Understanding patterns of bacterial diversity is of particular importance because bacteria may well comprise the majority of earth’s biodiversity and mediate critical ecosystem processes (Cavigelli and Robertson 2000; Torsvik et al. 2002).

## Polyphasic taxonomy: methods of studying microbial diversity

**Fig. 1:**
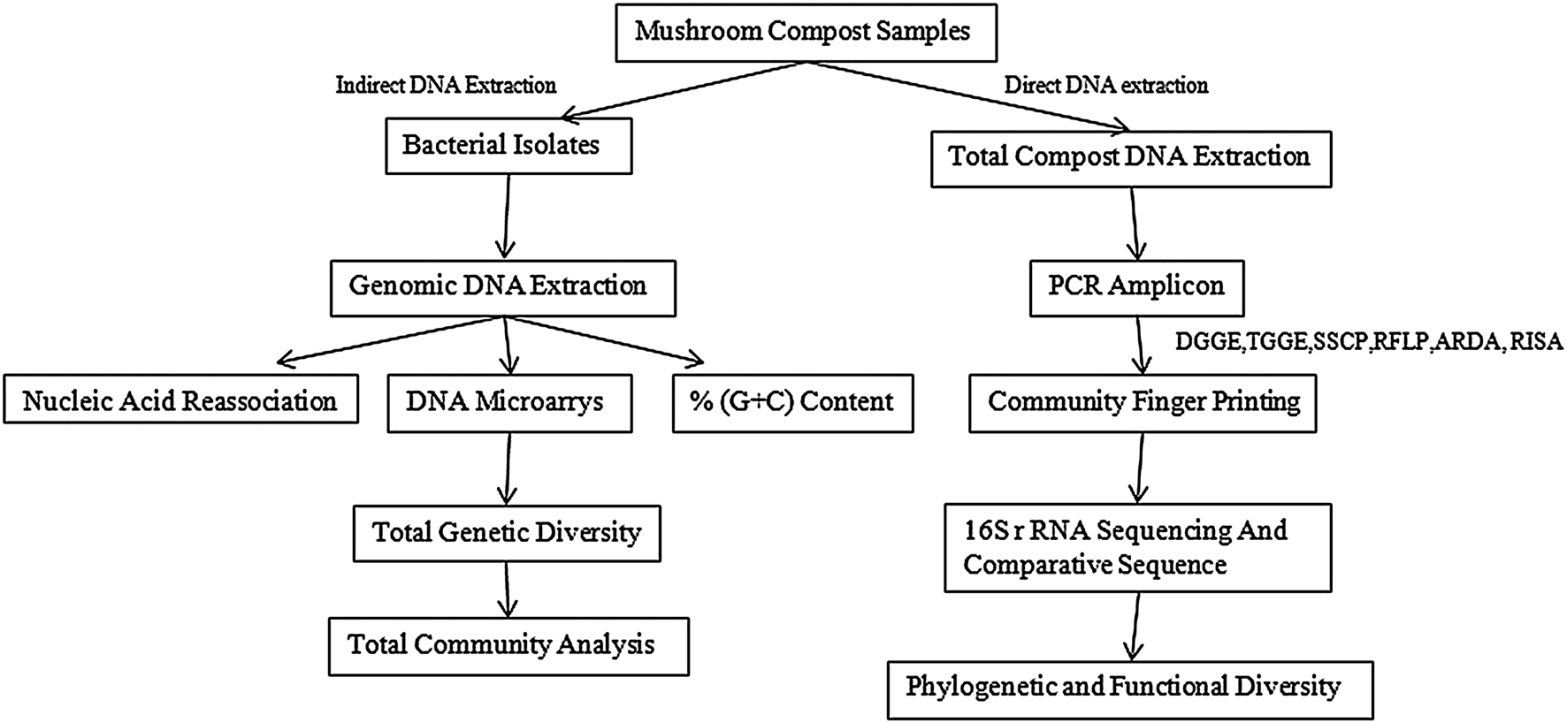
Molecular approach for analyzing microbial diversity

Taxonomy is generally taken as a synonym of systematic or biosystematics and is traditionally divided into three parts: (1) classification, i.e., the orderly arrangement of organisms into taxonomic groups on the basis of similarity; (2) nomenclature, i.e., the labeling of the units defined in (1), and (3) identification of unknown organisms, (Staley and Krieg 1984), i.e., the process of determining whether an organism belongs to one of the units defined in (1) and labeled in (2). According to Vandamme et al. (1996), all genotypic, phenotypic and phylogenetic information may be incorporated in polyphasic taxonomy (Fig. 1). Species diversity consists of species richness, the total number of species present, species evenness, and the distribution of species (Trevors 1998a, b; Overeas 2000). Methods to measure microbial diversity in natural environment can be categorized into two groups—phenotypic-based approaches and molecular-based approaches. It is difficult with current techniques to study true diversity since we do not know what is present and have no way of determining the accuracy of extraction or detection methods. The following review discusses in detail various molecular techniques and strategies for analysis of microbial diversity in mushroom compost. However, a brief summary of the phenotypic methods has been included along with their merits and demerits for comparison with the molecular methods, and to elucidate significance of the modern-day approaches.

### Phenotypic-based approaches for analyzing microbial diversity

#### Plate count

Traditionally, diversity was assessed using the selective plate method by Boulter et al. (2002) coupled with viable counts. Direct counting by fluorescent microscopy is reported to give 100–1000 times more than the number obtained by plate counting (Johnsen et al. 2001). These methods provide information on the active, heterotrophic component of the population (Trevors 1998a, b).

#### Carbon source utilization profile/community level physiological profile (CLPP)/BIOLOG

Community level physiological profile is a culture-dependent method of analyzing microbial communities. This technique takes advantage of traditional methods of bacterial taxonomy in which bacterial species are identified based on their utilization of different carbon sources. CLPP has been fascinated by the use of BIOLOG which is now widely available to assess functional diversity of microorganisms in compost ecosystem (Garland and Mills 1991).

#### Fatty acid methyl ester (FAME) analysis

Phospholipid fatty acid (PLFA) analysis has been used as a culture-independent method for assessing the structure of microbial community. Determination of phospholipid fatty acid (PLFA) profiles provides a broad diversity measurement of microbial community at the phenotypic level (Chayani et al. 2001). This method provides information on the microbial community composition based on grouping of fatty acids. Fatty acids are used as a chemotaxonomic marker. This is a signature molecule which is present in all living cells. In microorganisms, phospholipids found in cell membrane are the key determinant for this purpose. Fatty acids make up a relatively constant proportion of the cell biomass and can differentiate major taxonomic groups within the community. Therefore, change in the fatty acid profile can represent change in the microbial population in an environmental sample (Eiland et al. 2001) (Table 1).

**Table 1:** Merits and demerits of microbiological/biochemical methods to study microbial diversity from mushroom compost ecosystem

Method	Merits	Demerits
Community level physiological profiling (CLPP) Classen et al. (2003), Garland (1996), Garland and Mills (1991)	• Fast • Relatively inexpensive • Highly reproducible • Differentiate between microbial communities • Site-specific carbon sources can be used for the study	• Only represents culturable fraction of community • Represents only those organisms capable of utilizing available carbon sources • Represents metabolic diversity rather than microbial diversity • More suitable for fast-growing organisms
Fatty acid methyl ester analysis (FAME) Graham et al. (1995), Siciliano and Germida (1998), Zelles (1999)	• Direct extraction from soil can be done • Specific organisms or communities are followed • No culturing of microorganisms required	• Large amount of raw material is required in case of fungal spores • Can be influenced by external factors
Plate counts Tabacchioni et al. (2000), Trevors (1998b)	• Fast • Inexpensive • Ease of handling	• Unculturable microorganisms not detected • More suitable for fast-growing and non-fastidious bacteria • Fungi producing large amount of spores overgrow other microbes

### Molecular-based approach for analyzing microbial diversity

**Fig. 2:**
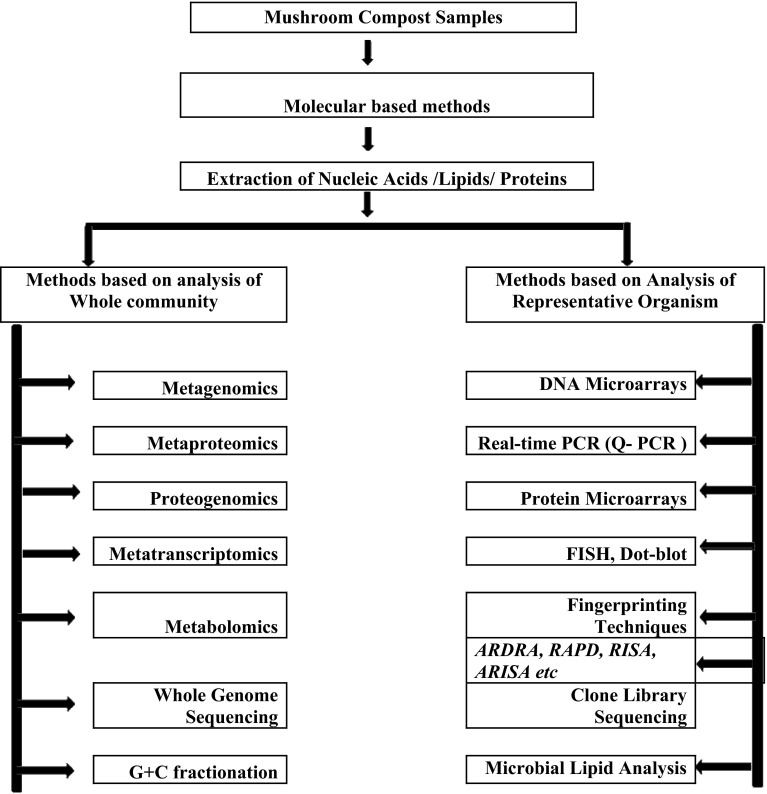
Culture-independent methods for characterization of microbial diversity from mushroom compost ecosystem

Traditional cultivation techniques for the enrichment and isolation of microbes yield only a limited fraction of all microorganisms present. Polymerase chain reaction (PCR)-based molecular methods provide a fast and sensitive alternative to conventional culture techniques. Molecular methods are based on the analysis of single cells, opening an opportunity to analyze the microbial community in its full diversity. To study population structures and dynamics, genetic fingerprinting techniques are required (Fakruddin et al. 2013). To understand the diversity and community composition of natural environment microflora, different molecular approaches have been developed by taxonomists that allow rapid profiling of microbial communities and provide information about specific phylogenetic groups present. PCR-based fingerprinting methods of microbial communities consist of:

1. first, the extraction of nucleic acids
2. second, the amplification of rRNA/rDNA, and
3. third, the analysis of PCR products by fingerprinting techniques (Fig. 2).

#### PCR-based approaches

Initially, molecular approaches for ecological studies relied on cloning of target genes isolated from environmental samples (DeSantis et al. 2007). PCR-based 16S rDNA profile provides information about prokaryote diversity and allows identification of prokaryotes as well as the prediction of phylogenetic relationships (Pace 1996, 1997, 1999). Therefore, 16S rDNA-based PCR techniques such as denaturing gradient gel electrophoresis (DGGE), temperature gradient gel electrophoresis (TGGE), single-strand conformation polymorphisms (SSCPs), amplified ribosomal DNA restriction analysis (ARDRA), terminal restriction fragment length polymorphisms (T-RFLPs) and ribosomal intergenic spacer analysis (RISA). can provide detailed information about community structure of an ecosystem in terms of richness, evenness and composition and can be used to compare different species present in a sample such as compost (Rawat and Johri 2014).

#### Amplified ribosomal restriction DNA analysis (ARDRA)

Amplified ribosomal DNA restriction analysis (ARDRA) is based on DNA sequence variations present in PCR-amplified 16S rRNA genes (Smit et al. 1997). The PCR product amplified from environmental DNA is generally digested with tetracutter restriction endonucleases (e.g., *Alu*I and *Hae*III), and restricted fragments are resolved on agarose or polyacrylamide gels. Although ARDRA provides little or no information about the type of microorganisms present in the sample, the method is still useful for rapid monitoring of microbial communities over time, or to compare microbial diversity in response to changing environmental conditions. ARDRA is also used for identifying the unique clones and estimating OTUs in environmental clone libraries based on restriction profiles of clones (Smit et al. 1997)

Amplified ribosomal DNA restriction analysis (ARDRA) is used to study the microbial diversity that relies on DNA polymorphism. The technique of amplified ribosomal DNA restriction analysis (ARDRA) is based on repetitive units of nuclear ribosomal DNA (rDNA) consisting of conserved coding and variable non-coding regions. The coding and non-coding regions are amplified by PCR, the amplicon is digested by restriction endonucleases, and the restriction fragments are separated according to their size using gel electrophoresis. Liu et al. (1997) also have reported that PCR-amplified 16S rDNA is digested with a 4-base pair cutting restriction enzyme. Pace (1999) has reported that gel electrophoresis banding patterns can be used to screen clones and used to measure bacterial community structure. It has been reported that this is useful for detecting structural changes in microbial communities but not as a measure of diversity or detection of specific phylogenetic groups (Liu et al. 1997). ARDRA is a sensitive technique giving high resolution to provide reliable genotypic characterization at the community level of compost bacteria (Heyndrickx et al. 1996). This technique has frequently been used to understand community structure from a variety of samples such as feathers (Tiquia et al. 2005), bacteria present in the self-heating phase of composting material (Koschinsky et al. 1999), and bacteria present in the casing (Choudhary et al. 2009; Choudhary 2011).

ARDRA-ITS (also termed ITS-RFLP) uses the universal primers ITS 1 and ITS 4 (White et al. 1990) which anneal to the evolutionary stable 18S and 28S rRNA genes. This attachment with conserved rDNA regions allows the investigation of fungi without prior knowledge of their genome organization. The conserved domains are interrupted by the non-coding variable internal transcribed spacers ITS I (between 18S and 5.8S) and ITS II (between 5.8S and 28S) which are informative for differentiation. ARDRA-ITS exhibits differences at the species and subspecies levels. Studies on indoor basidiomycetes are rare. In T-RFLP, the stepwise strategy is to extract DNA from bacterial communities, and use it as a template for PCR amplification of desired gene(s); 16S rDNA is routinely used for compost microbes, with appropriate primers. The amplified DNA is digested with restriction enzymes and the size of terminal fragment generated by each amplicon is used for the estimation of bacterial community structure of the compost without the need of sequencing the terminal fragments (Choudhary et al. 2009). The abundance and the size distribution of DNA fragments are used to estimate the genetic diversity of bacterial community. However, this method is not pertinent for the estimation of phylogenetic positions of community members. T-RFLP analysis of microbial community during composting revealed extensive bacterial diversity. Molecular tools for the identification of casing soil bacteria were used and 16S rRNA gene analysis was intensively used to understand the phylogenetic relationships (Choudhary et al. 2009). For the 16S rRNA gene analysis, amplified ribosomal DNA restriction analysis (ARDRA) was performed. This molecular technique has been successfully used for bacterial community analysis in a great variety of environments. Fifty casing soil isolates, representing different cropping stages/environmental niches (source location) including endotrophs, were selected based on colony morphology and were subjected to ARDRA analysis by the digestion of amplified 16S rRNA gene with *Taq*I, *Msp*I, and *Alu*I. The dendrogram of banding patterns was obtained after combination of three independent digestions. The similarity value ranged widely between 50 and 100 %. The first group was represented by reference strains, viz., biovars of *P. fluorescens* I–V that showed 100 % similarity, whereas reference strain *P. chlororaphis* showed ~60 % similarity with biovars of *P. fluorescens*. Most groups showed 90–100 % similarity with some groups delineated at 80–90 % similarity level. This study examined the culturable bacterial community comprising successive stages of casing soils and endotrophs using ARDRA and 16S rRNA gene fragment sequencing. Results show considerable diversity of bacterial community in casing soils based on the large number of ARDRA patterns obtained. Phylogenetic analysis revealed that 85 % of the bacterial isolates belonged to y-proteobacteria group and other isolates were bacilli. A single isolate in this study was found to belong to the genus Sphingobacterium. Two genera, Acinetobacter and Pseudomonas were dominant and were sole representative of y-proteobacteria. Dominance of the genus acinetobacter was of significance since this had not been reported earlier from the mushroom casing ecosystem (Choudhary et al. 2009). A total of 46 bacterial isolates, representing different stages of mushroom compost viz., pre-wetting, filling, pasteurizing, conditioning, spawning and drenching, were selected on the basis of structural and functional potentials (Agrawal et al. 2011) and were subjected to ARDRA analysis by the digestion of amplified 16S rDNA gene with *Rsa*I, and *Hae*III. Dendrogram pattern obtained after the combination of three independent digestions showed 11 groups. The similarity value ranged widely between 30 and 100 %. There were Groups 2, 4, 7, 9 and 10th that showed 100 % similarity among respective isolates. Most groups showed 90–100 % similarity but some of them delineated at 70–90 % similarity level.

#### Random amplification of polymorphic DNA (RAPD)

The analysis of randomly amplified polymorphic DNA (RAPD) is based on the polymerase chain reaction (PCR) using short (about 10 bases) randomly chosen single primers which anneal as reverted repeats to the complementary sites in the genome (Agrawal and Shrivastava 2013). The DNA between the two opposite sites with the primers at starting and end points is amplified by PCR. The amplification products are separated on gels, and the banding patterns distinguish organisms according to the presence/absence of bands (polymorphism). It is a peculiarity of RAPD analysis that it discriminates at different taxonomical levels, viz., isolates and species, depending on the fungus investigated and the primer used. Random amplification of polymorphic DNA (RAPD) is a technique that makes use of a single random primer at low stringency for the amplification of polymorphic DNAs (Williams et al. 1990). The primer at low stringency anneals to the target DNA at different sites whose sequences may not be exactly complementary to the primer sequence. Several discrete DNA bands are amplified upon annealing of primer in inverted orientation at distances suitable for amplification. Although RAPD analysis is rapid and convenient it is not reproducible, even small variations in the batch of Taq polymerase or buffer may change the fingerprint. The conditions for the direct amplification of DNA in natural habitats by RAPD techniques therefore must be optimized case-by-case, to take advantages and usefulness of the technique. RAPD analysis of ITS region of 5.8S rRNA gene from eight *H*. *grisea* isolates was carried out by (Singh et al. 2005). Intra-specific diversity was visualized in this region. Isolates within these species exhibit genetic differences which were correlated with morphological variation. The genetic variation exhibited by *Torula*–*Humicola* complex has drawn considerable attention. Straatsma and Samson (1993) studied the genetic diversity among *S. thermophilum* isolates using RAPD analysis and found that these exhibited distinct amplification pattern.

#### REP-PCR (BOX element)

Another method to obtain genomic DNA fingerprint of bacteria is the repetitive extragenic palindromic-PCR (rep-PCR). This PCR uses primers to match short consensus repetitive sequences. Three different primers can be used namely BOX (originally described in *Streptococcus pneumoniae*), ERIC (originally described in *Salmonella typhimurium*) and REP (originally described in *Escherichia coli*) (Gomez et al. 2000). Differences in band sizes represent polymorphism in the distance between the repetitive elements of different strains. Repetitive extragenic palindromic-PCR (rep-PCR) is a genotypic method that uses oligonucleotide primers complementary to repetitive sequences dispersed throughout the genome of *E. coli* (Versalovic et al. 1991). Using PCR, this method amplifies diverse regions of DNA flanked by ‘rep’ sequences, leading to amplicon patterns specific for an individual *E. coli* strain (Rademaker and de Bruijn 1997). The conserved repetitive sequences are divided into four types: the repetitive extragenic palindromic (REP) sequences, the enterobacterial repetitive intergenic consensus (ERIC) sequences, the BOX sequences and the polytrinucleotide (GTG) 5 sequences (Versalovic et al. 1994). Five rep-PCR methods, such as REP-PCR (primer sets Rep1R-I and Rep2-I); ERIC-PCR (primer sets ERIC1R and ERIC2); ERIC2-PCR (primer ERIC2); BOX-PCR (primer BOX A1R); and (GTG)5-PCR [primer (GTG)5], are commonly used for genotyping of different bacterial strains. The BOX-PCR is the multilocus analysis and produces higher degree of resolution among the isolates. The repetitive sequences in the form of BOX elements are randomly located within the whole genome and the BOX primers amplify genomic regions between the two BOX elements. Selenska-Pobell et al. (1995) reported distribution of these repetitive sequences (BOX and ERIC) as nearly a true reflection of genomic structure and amplification of inter-REP elements often detects similarities in a given group of bacteria. It is anticipated that REP- and ERIC-like sequences are virtually ubiquitous in bacteria and facilitate rapid molecular characterization by PCR-based fingerprinting (Versalovic et al. 1991). REP-PCR has been used for the identification of bacteria since it provides genomic fingerprint of chromosome structure which is considered variable between strains (Choudhary et al. 2009). Both prokaryotic and eukaryotic genomes contain dispersed repetitive sequences that range from 15 to several hundred base pairs in length. These elements are non-coding but are present in high copy number relative to the longer repeated elements, which contain coding sequences. The interspersed repetitive sequences described in bacteria are BOX elements (154 bp), Rep sequences (386 bp) and ERIC sequences (124 bp). These sequences may be diagnostic and allow differentiation down to the species or strain level. All bacterial isolates recovered from different stages of mushroom composting were subjected to REP-PCR analysis by BOX primer. A distinctive banding pattern was observed in the BOX element. The dendrogram of banding patterns based on UPGMA showed significant discriminatory relationship among the isolates. A total of 14 groups were formed for the 46 isolates used with 30–100 % similarity. Isolates placed within nine groups showed 100 % similarity within any single group; four groups showed 37–70 % similarity among respective isolates. Two groups were quite distinct from other groups since they had no similarity with other groups.

#### Ribosomal intergenic spacer analysis (RISA)/automated intergenic spacer analysis (ARISA)

RISA and ARISA provide riobosomal-based fingerprinting of the microbial community. In RISA and ARISA, the intergenic spacer (IGS) region between 16S and 23S ribosomal subunits is amplified by PCR, denatured and separated on polyacrylamide gel under denaturing conditions. IGS region may encode tRNAs and is useful for differentiating between bacterial strains and closely related species because of the heterogeneity of IGS length and sequence (Ranjard et al. 2000; Fisher and Triplett 1999). RISA provides a community-specific profile, with each band corresponding to at least one organism in the original community. The automated version of RISA is known as ARISA and involves use of a fluorescence-labeled forward primer, and ISR fragments are detected automatically by a laser detector. ARISA allows simultaneous analysis of many samples; however, the technique has been shown to overestimate microbial richness and diversity (Fisher and Triplett 1999). 16S rRNA sequences are highly conserved among eubacteria (Woese 1987) and analysis of genetic variation in this region is not appropriate to differentiate strains within the species. Ribosomal Intergenic Sequence Analysis (RISA) is also used for analyzing the species composition in compost ecosystem (Saison et al. 2005). A total change in fungal community structure in the initial stage of composting was characterized by employing F-ARISA and 18S rRNA gene cloning and sequencing (Hansgate et al. 2004). This technique involves the analysis of polymorphism of the length of intergenic spacer between rrs (16S rRNA) and rrl (23S rRNA) genes, whose sizes may vary from 50 bp to more than 1.5 kb depending on the species. The subsequent sequencing of amplicons can also allow taxonomic identification of specific populations within a community.

#### Denaturing gradient gel electrophoresis (DGGE)/temperature gradient gel electrophoresis (TGGE)

DGGE examines microbial genetic diversity based on the electrophoresis of PCR-amplified 16S rDNA fragments in compost (Muyzer et al. 1993). DGGE and TGGE were first developed to detect point mutation in DNA sequences. DNA is extracted from samples and amplified using PCR with universal primers targeting part of the 16S or 18S rRNA sequences. The 5′-end of forward primer contains a 35–40 base pair GC clamp to ensure that at least some part of DNA remains double stranded. Separation on a polyacrylamide gel with a gradient of increasing concentration of denaturants (formamide and urea) will occur based on the melting behavior of double-stranded DNA. Upon denaturation, DNA melts in domains, which are sequence specific and will migrate differentially through the polyacrylamide gel. Molecules with different sequences may have a different melting behavior, and will, therefore, stop migrating at different positions in the gel (Muyzer and Smalla 1998). After this, DNA bands in DGGE/TGGE profiles can be visualized using ethidium bromide, SYBR Green I, or silver staining, which are more sensitive than others but also stain single-stranded DNA; however, which can be digested by nuclease to reduce the interference. DGGE/TGGE have been used widely in environmental microbiology to study community complexity, monitor population shifts, analyze enrichment cultures, isolate bacteria, detect sequence heterogeneity of 16S rRNA genes/18S rRNA, compare DNA extraction methods, screen clone libraries and determine PCR and cloning biases (Muyzer and Smalla 1998; Nicolaisen and Ramsing 2002). DGGE is used to detect non-RFLP polymorphism (Bodelier et al. 2000). This technique utilizes sequence variations in PCR-amplified DNA fragments of identical length and resolves them on the basis of differences in their mobility in polyacrylamide gels containing gradient of a denaturing agent (Muyzer et al. 1993). Initially, the fragments move according to molecular weight, but as they progress into higher denaturing conditions, each (depending on its sequence composition) reaches a point where DNA begins to melt. This has been proven to be a valuable approach to obtain 16S rRNA gene profiles to identify temporal and spatial differences in bacterial community structure in compost ecosystem (Takaku et al. 2006; Ishii et al. 2000). When DGGE analyses of rRNA genes are combined with hybridization using phylogenetic probes or with sequencing, assessment of the phylogenetic affiliation of numerically dominating members of community can be obtained. DGGE amplified 16S rDNA fragments have been used to analyze microbial succession during the laboratory-scale composting process of garbage (Ishii et al. 2000). Kowalchuk et al. (1999) detected beta subgroup proteobacterial ammonia oxidizer like sequences in the commercial mushroom compost by separating the product of PCR and RT-PCR by DGGE and identifying them by hybridization with hierarchial set of oligonucleotide probes designed to detect ammonia oxidizer like sequence cluster in genera *Nitrospira* and *Nitrosomonas.* The succession and phylogenetic profile of eukaryotic communities in the composting process of rice straw were studied employing DGGE followed by 18S rDNA (Cahyani et al. 2003).

Instead of using a gradient of the denaturant, TGGE uses a uniform concentration of denaturant in the gel and temperature is increased uniformly with time throughout the electrophoresis. This may result in more easily reproducible separations than those commonly obtained with DGGE. The most active bacteria are detected by TGGE of the rRNA amplicon obtained by RT-PCR (Felske et al. 1998). Bruns et al. (1999) have determined spatial and temporal diversity of ammonia oxidizers in native, tilled and successional soils using DGGE and TGGE. The advantage of DGGE and TGGE compared with T-RFLP is that each amplicon (band) within a bacterial community profile can be isolated and characterized by DNA sequencing. Thus, samples can be compared not only by their profile patterns but selected components can also be clearly identified. But, DGGE can only determine the microbes that constitute up to 1 % of the total bacterial community (Zoetendal et al. 2004). Therefore, the separation of amplicons by DGGE may not be perfect and amplifying-based sequence analyses need careful interpretation (Nikolausz et al. 2004).

#### Single-strand conformation polymorphisms (SSCPs)

Single-strand conformation polymorphism (SSCP) analysis is a method used for detecting sequence differences of single-stranded DNA (ssDNA) by non-denaturing polyacrylamide gel electrophoresis (PAGE). In general, the SSCP process involves PCR amplification of the target DNA, denaturation of the double-stranded PCR product with heat and formamide (or other denaturants), and sample resolution by non-denaturing PAGE (Orita et al. 1989). During electrophoresis, ssDNA fragments are expected to fold into a three-dimensional shape depending mainly on their primary sequence. Several authors have suggested that even if the difference in the sequence between the wild-type sample and a mutated fragment is just a single nucleotide, a unique and distinct electrophoretic mobility pattern will be adopted by each sequence. Therefore, complex mixtures of DNA species of the same size can be separated by non-denaturing PAGE into bands of different mobilities, due to a difference in their predominant semi-stable conformations. SSCP-PCR was developed to detect novel polymorphisms or point mutations in DNA (Orita et al. 1989). SSCP is a technique to distinguish DNA molecules of the same size but of different nucleotide sequences using gel electrophoresis in non-denaturing polyacrylamide gels due to differences in mobility caused by their folded secondary structure. PCR product of the same size but different base sequences can be distinguished by SSCP, which makes this method a promising tool for the analysis of compost microbial community at the ribosomal gene level (Rawat et al. 2005). The diversity of bacterial and fungal communities in the compost was analyzed by single-strand conformation polymorphisms (SSCPs) of approximately 400 bp PCR products, which were amplified with universal primer for 16S rRNA (bacteria) and 18S rRNA (fungi), with compost DNA as a template. The generated pattern showed succession of different members of microbial community during self-heating phase (Peters et al. 2000). Community succession during 18-day-long mushroom composting revealed the presence of lactobacilli at early stage of composting (Peters et al. 2000). This technique is an alternative and possibly an improvement over DGGE and TGGE. SSCP does not require the construction of gradient gels thereby increasing the reproducibility of gels. For TGGE, specific equipment is needed, a temperature gradient incubation system for electrophoretic gels. For SSCP, regular electrophoretic chambers with temperature control can be used. Another advantage of SSCP compared with DGGE/TGGE is that compatible primers for SSCP are easier to design, since consideration regarding the primer bias for the formation of a GC clamp during the PCR process is not required (Droffner and Brinton 1995).

#### PCR-independent approaches

##### Guanine plus cytosine (G + C) content

Difference in the guanine plus cytosine (G + C) content of DNA is used to measure bacterial diversity of compost. Tiedje et al. (1999) have reported that microorganisms differ in their G + C content and that taxonomically related groups differ only by 3 and 5 % (Nusslein and Tiedje 1999). Thus, the fractionation of total community DNA can be achieved by density gradient centrifugation based on G + C content. The technique generates a fractionated profile of the entire community DNA and indicates relative abundance of DNA (hence taxa) as a function of G + C content. The total community DNA is physically separated into highly purified fractions, each representing a different G + C content that can be analyzed by additional molecular techniques such as DGGE/ARDRA to better assess total community diversity. It provides a coarse level of resolution as different taxonomic groups may share the same G + C content. G + C analysis is not influenced by PCR biases and since it includes all extracted DNA, and uncovers rare members in microbial population.

#### DNA-reassociation kinetics and DNA:DNA hybridization

Whole-genome DNA–DNA hybridization (DDH) offers true genome-wide comparison between organisms. A value of 70 % DDH was proposed as a recommended standard for bacterial species delineation (Goris et al. 2007). Typically, bacterial species having 70 % or greater genomic DNA similarities usually have >97 % 16S rRNA gene sequence identity. Although DDH techniques have been originally developed for pure culture comparisons, they have been modified for use in whole microbial community analysis. In DDH technique, total community DNA extracted from an environmental sample is denatured and then incubated under conditions that allow them to hybridize or reassociate.

Nucleic acid hybridization is a process wherein two DNA or RNA single chains (mono-stranded) from different biological sources form a double catenary configuration, based on contingent sequence homology between two sources, resulting in DNA–DNA, RNA–RNA or DNA–RNA hybrids. The purpose is identification or localization of certain nucleic acid sequences (genes) in the genome of some species. Two basic notions are used: the target molecule representing the DNA, RNA or protein sequence that has to be identified and the probe molecule that identifies the target, by hybridization. When hybridization takes place on a solid carrier, it is named as blotting and is divided in three categories:

- Southern blotting where DNA molecules are identified using DNA or RNA probes;
- Northern blotting where RNA molecules are identified using RNA or DNA probes;
- Western blotting where protein sequences are identified using specific antibodies.

DNA reassociation is used to measure genetic complexity of the microbial community and has been applied to evaluate environmental diversity. Total DNA is extracted from the environmental samples, purified, denatured and allowed to reanneal. The rate of hybridization or reassociation will depend on the similarity between DNA sequences. (Theron and Cloete 2000) have reported that as the complexity of diversity in DNA sequences increases, the rates at which DNA reassociates decrease. Similarity between communities of two different samples can be studied by measuring the degree of similarity between DNA through hybridization kinetics (Griffiths et al. 1999). Nucleic acid hybridization using specific probes is an important qualitative and quantitative tool in molecular bacterial ecology (Clegg et al. 2000; Theron and Cloete 2000). This approach can be applied on extracted DNA, or RNA, under in situ conditions. Oligonucleotide or polynucleotide probes designed from known sequences ranging in specificity from domain to species can be tagged with fluorescent markers at the 5′ end (Theron and Cloete 2000). However, dot blot hybridization is used to measure the relative abundance of a certain group of microorganisms. It provides valuable spatial distribution information on microbial community or environmental samples to which microbial community similarity is compared.

Broad-scale analysis of community DNA, using techniques such as DNA-reassociation kinetics, provides information about the total genetic diversity in compost microbial community (Torsvik et al. 2002). This approach is based on the assumption that more complex denatured DNA reassociates at a slower rate than less complex denatured DNA, and that the kinetics of reassociation is proportional to the genomic complexity. The advantage of this approach is that it may be the only means developed to date by which total number of bacterial species within a compost sample can be determined. Requirement of large quantity of DNA in this technique is the main limitation because it is technically difficult to obtain high DNA yield from soil sample. DNA:DNA hybridization provides a reasonably good means of comparing two microbial communities, although it may suffer from the lack of sensitivity that makes quantitative comparison of communities possessing similar structures difficult. Similarly, shift in GC content can be used to detect changes in bacterial community but does not provide any information regarding richness (number of species), evenness (relative abundance) and composition of the microbial community (Torsvik et al. 2002).

#### DNA microarrays

DNA microarrays have been used primarily to provide a high-throughput and comprehensive view of microbial communities in environmental samples. The PCR products amplified from total environmental DNA are directly hybridized to known molecular probes, which are attached on the microarrays (Gentry et al. 2006). After the fluorescently labeled PCR amplicons are hybridized to the probes, positive signals are scored by the use of confocal laser scanning microscopy. The microarray technique allows samples to be rapidly evaluated with replication, which is a significant advantage in microbial community analyses. In general, the hybridization signal intensity on microarrays is directly proportional to the abundance of the target organism. The latest development includes application of DNA microarrays to detect and identify bacterial species or to assess microbial diversity (Cho and Tiedje 2001; Green and Voordouw 2003). This rapidly characterizes the composition and functions of microbial communities because a single array contains thousands of DNA sequences with a possibility of very broad hybridization with wide identification capacity. The microarrays can contain specific target genes such as nitrate reductase, nitrogenase or naphthalene dioxygenase to provide functional diversity information or can contain ‘standard’ of environmental samples (DNA fragments with less than 70 % hybridization) representing different species found in the environmental sample (Green and Voordouw 2003).

#### Reverse sample genome probing (RSGP)

RSGP is used to analyze microbial community composition of the most dominant culturable species. RSGP includes four steps: (1) isolation of genomic DNA from pure cultures, (2) cross-hybridization testing to obtain DNA fragments with less than 70 % cross-hybridization, (3) preparation of genome arrays on a solid support, and (4) random labeling of a defined mixture of total community DNA and internal standard. RSGP is a useful approach when diversity is low, but several molecular biologists face difficulty while assessing community composition of diverse habitats (Green and Voordouw 2003) (Table 2).

**Table 2:**
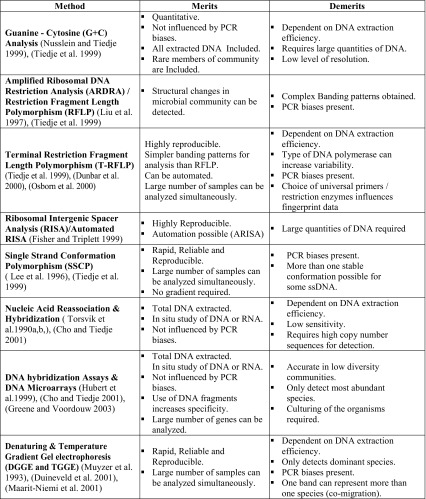
Merits and demerits of molecular-based methods to study microbial diversity from mushroom compost ecosystem

#### Phylogenetic analysis and 16S rDNA gene sequencing

Taxonomy based on comparative phylogenetic analysis of 16S rRNA genes, first introduced by Carl Woese, presents a radical departure from classical taxonomy. Cellular life forms could be divided into three primary phylogenetic domains: Archaea, Bacteria and Eucarya (Woese et al. 1990; Olsen et al. 1986). For microorganisms, molecular data often provide considerable information because microorganisms such as bacteria simply do not have the diversity of form to make morphological characteristics useful in establishing phylogeny. Aside from derivation of taxonomies, phylogenetic analyses are important in identifying similarity between organisms, providing an ability to understand physiology and ecology of non-culturable species. PCR-based 16S rDNA profile provides information about microbial diversity and allows identification of microbes and prediction of phylogenetic relationships (Pace 1997; Song et al. 2001). Initially, molecular approaches for ecological studies relied on cloning of target genes isolated from environmental samples (Muyzer and Smalla 1998). Therefore, 16S rDNA-based PCR techniques such as DGGE, TGGE, SSCP, ARDRA, T-RFLP, RISA and others can provide detailed information about community structure of various ecosystems in terms of diversity, invariability and constitution and can be used to compare species present in mushroom compost. Nucleic acid sequencing provides larger discrimination than other methods and better characterization of a particular member of community (McCaig et al. 2001). 16S rRNA sequences have been used most extensively to classify the biodiversity. The difference in sequences can be used to construct a phylogenetic tree (Swofford et al. 1996). The phylogenetic approach for the systematic assessment of culturable microbial diversity up to the taxonomic level using nucleic acid hybridization and 16S rDNA sequences analysis has been of immense utility in the phylogenetic reformation of the classification of prokaryotic organisms (Woese 1987). A total of 40 isolates out of 46 were selected for 16S rDNA partial sequencing. Blast database of NCBI was used to compare the sequence of compost isolates with the known 16S rDNA sequences in the existing data base to search for homogenous sequences in the gene bank (Altschul et al. 1997). The result obtained was confirmed by comparing the information with ribosomal database project. The ClustalW programme, from the European Bioinformatics Institute (EMBL), was used to align the sequences through BLAST (http://www.ncbi.gov/BLAST), FASTA (http://www.ebi.ac.uk/FASTA 31), and ClustalW (http://www.ebi.ac.uk/ClustalW1). All the sequences obtained from 40 isolates were aligned with each other to determine genetic diversity between the bacteria, isolated from mushroom compost. Comparison of 16S rDNA sequence with reference strain viz., *A*. *baumannii* AY847284, *Alcaligene* AY346136, *A*. *arilaitensis* AY635865, *B*. *cereus* AB190065, *B*. *licheniformis* AJ717380, *B*. *pumilus* AY294325, *B*. *subtilis* AB190027, *B*. *vallismortis* AY484784, *Ochrobactrum* sp. AJ920029 and *S*. *maltophilia* AJ131781, to which they matched was performed. A consensus tree was drawn from aligned sequences using DNAMAN version 4.0. All isolates showed 49–100 % similarity among each other. On the basis of 16S rDNA sequence homology tree, two major clusters A and B at 67 % and one small cluster at 49 % similarity level were found (Agrawal et al. 2011).

A variety of bacilli were reported by from various phases of mushroom composting process utilizing a DNA sequence-based approach (Rawat et al. 2005). Dominant species included *Bacillus* *licheniformis* (AY871062), *Bacillus* *megaterium*, *Bacillus* *subtilis* (AY940671) and *Alcaligenes* sp. (AY871052) from the end of phase 1 compost; *Bacillus cereus* (AY871057) and *Bacillus subtilis* (AY871054) from peak heat stage; *Bacillus subtilis* (AY871059) from the end of phase II compost and *Bacillus pumilus* (AY864923) from drenching. Thus, the appearance and disappearance of species of *Bacillus* continued throughout the composting period. Other bacterial species sequenced were *Arthrobacter arilaiti* (AY871060) from the drenching phase. The total DNA or rRNA extraction from compost sample followed by 16S (Prokaryotes) and 18S (eukaryote) analyses does not however always reflect the presence of qualitative and quantitative diversity (Gurtner et al. 2000; Ishii et al. 2000).

## Conclusion

Microbial communities have great potential for temporal or spatial change, and represent a powerful tool for understanding community dynamics in ecological context. Variations in microbial community structure can influence ecosystem process. This study defines the complexity of microbial community dynamics and how it affects the ecosystem process. Application of polyphasic approach (phenotypic and genotypic component) of bacterial diversity, coupled to the utilization of phylogenetic analysis has permitted deeper insights at subtle changes that result in biological conditioning of compost to permit mushrooming and also to suggest where artificial inocula can be used to hasten the composting process and associated mushroom yield. Although methods to study diversity (numerical, taxonomic, and structural) are improving for both bacteria and fungi, a clear association between diversity and function is still not known. It is generally thought that a diverse population of organisms will be more resistant to stress and more capable of adapting to environmental changes. Besides the above, other tools have been used to characterize the whole microbial community of mushroom compost ecosystem to reveal microbial diversity and function.
